# Supplementary material for: Altered microRNA Transcriptome in Cultured Human Liver Cells upon Infection with Ebola Virus
Source: Int J Mol Sci. 2021 Apr 6;22(7):3792. doi: 10.3390/ijms22073792 (PMC8038836; doi:10.3390/ijms22073792)
Supplement: Supplementary file 1 [file ijms-22-03792-s001.zip › Supplementary_File/C_ GO_Analysis_Results/16-30nt_go_Mayinga-96h-Huh7_vs_Control-96h-Huh7_up.mature_mirna_targets/MF_EnrichmentScoreDotPlot.pdf]

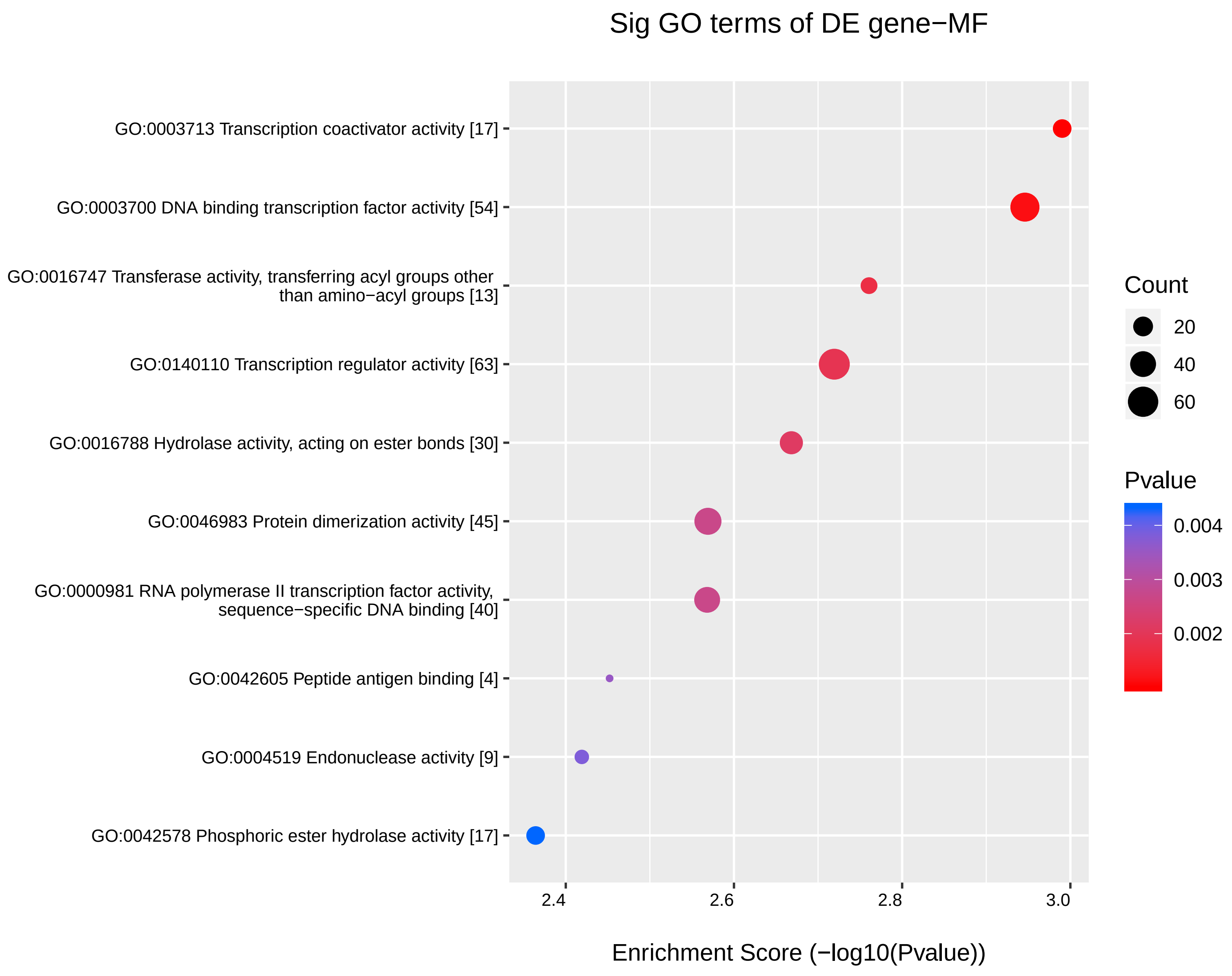

Sig GO terms of DE gene−MF
GO:0003713 Transcription coactivator activity [17]
GO:0003700 DNA binding transcription factor activity [54]
GO:0016747 Transferase activity, transferring acyl groups other than amino−acyl groups [13]
GO:0140110 Transcription regulator activity [63]
GO:0016788 Hydrolase activity, acting on ester bonds [30]
GO:0046983 Protein dimerization activity [45]
GO:0000981 RNA polymerase II transcription factor activity, sequence−specific DNA binding [40]
GO:0042605 Peptide antigen binding [4]
GO:0004519 Endonuclease activity [9]
GO:0042578 Phosphoric ester hydrolase activity [17]
2.4
2.6
2.8
3.0
Enrichment Score (−log10(Pvalue))
Count
20
40
60
Pvalue
0.004
0.003
0.002
